# Lipid transport and epithelial barrier integrity

**DOI:** 10.18632/oncotarget.5233

**Published:** 2015-08-22

**Authors:** Jill M. Fritz, Li Yang, Timothy E. Weaver

**Affiliations:** Division of Pulmonary Biology, Children's Research Foundation and Department of Pediatrics, University of Cincinnati College of Medicine, Cincinnati, OH, USA

**Keywords:** asthma, phospholipid transport protein, mitochondria, Stard7, START domain

Airway epithelial cells form a paracellular barrier through apical junctional complexes that physically separate the host from its external environment. This barrier serves several important functions including protection from harmful microorganisms and regulation of the bi-directional flow of ions and macromolecules across the lung epithelial barrier. Interestingly, several studies have recently reported structural and functional defects in airway epithelial cells of individuals with allergic disease [1]. Although environmental factors such as allergens, viruses and pollutants have been shown to damage the epithelial barrier and increase allergen sensitization, genetic factors also likely play a role in epithelial dysfunction of susceptible individuals. In support of this concept, a study recently published by our laboratory provides insight into a novel intracellular pathway associated with epithelial barrier integrity and susceptibility to allergic disease [2].

Microarray analyses revealed a reduction in expression of a lipid transport gene, Stard7, in nasal epithelial cells isolated from individuals experiencing an acute asthmatic exacerbation compared to those with stable asthma or nonatopic controls [3]. To further explore the significance of this gene in asthma, we generated mice globally deficient in Stard7. Although the majority of mice homozygous for the disrupted allele died at embryonic day 11 (likely of cardiovascular abnormalities), heterozygous mice survived and exhibited increased inflammation, mucous cell metaplasia and airway hyperresponsiveness in a common model of experimental asthma. Importantly, following exposure to antigen, lung epithelial barrier permeability was significantly increased in haploinsufficient mice compared to wild type littermates. These findings were associated with increased activation of dendritic cells, providing further support for the concept that altered barrier integrity increases allergen penetrance and immune cell activation. Further, as Stard7 heterozygous mice aged, they developed spontaneous dermatitis, a chronic inflammatory disorder of the skin, under sterile living conditions [2]. Collectively, these data suggested that Stard7 functions in a unique protective pathway in tissues exposed to the extracellular environment.

Stard7 belongs to the START (steroidogenic acute regulatory protein-related lipid transfer) domain superfamily that facilitates transfer of lipids among intracellular membranes. Three members of the PCTP/Stard2 subfamily of START domain proteins, Stard2, Stard7 and Stard10, all bind and transport phosphatidylcholine (PC). Stard7 was previously shown to promote uptake of PC by mitochondria [4-5]. Mitochondria are incapable of synthesizing PC, a phospholipid that comprises up to 45% of total phospholipid in this organelle. Given the importance of PC import for mitochondrial integrity/homeostasis, there is likely some redundancy in this pathway. Consistent with this hypothesis, Stard2 was also implicated in trafficking of PC to mitochondria [4]. It would therefore be of considerable interest to determine if loss of Stard2 or Stard10 (not yet shown to transport PC to mitochondria) expression also predispose to allergic disease.

Mitochondria perform several essential cellular functions including ATP production, calcium regulation and redox homeostasis. Mitochondrial dysfunction often leads to increased generation of reactive oxygen species (ROS) and importantly, recent studies have shown that mitochondrial-derived ROS increased epithelial barrier permeability in a mouse model of colitis [6]. Polymorphisms in the mitochondrial genome are also associated with the development of asthma in humans, and mitochondrial swelling and dysfunction in the airway epithelium has been linked to asthma pathogenesis [7]. Collectively these observations suggest a model in which decreased expression of Stard7 (and perhaps other PC transporters) leads to mitochondrial dysfunction and a subsequent increase in epithelial barrier permeability that promotes allergen sensitization. Although speculative, PC transport may also influence immune tolerance by an as yet unidentified molecular pathway(s).

Overall, this study revealed an important role for intracellular lipid transport in epithelial barrier function and immune homeostasis. Further studies are underway to determine if Stard7 deficiency in epithelial cells affects mitochondrial function and formation of apical junctional complexes in the airway epithelium. Understanding the molecular mechanisms regulating epithelial barrier integrity could lead to novel therapeutics for the treatment of allergic airway disease.
